# Closure of long‐standing oroantral fistula: Surgical challenge in medically compromised patient—A case report

**DOI:** 10.1002/ccr3.3051

**Published:** 2020-06-23

**Authors:** Arvind Ramanathan, Vishak Acharya

**Affiliations:** ^1^ Department of Oral & Maxillofacial Surgery Manipal College of Dental Sciences Mangalore Karnataka India; ^2^ Manipal Academy of Higher Education Manipal Karnataka India; ^3^ Department of Pulmonary Medicine Kasturba Medical College Mangalore Karnataka India

**Keywords:** buccal advancement flap, buccal fat pad, maxillary sinusitis, oroantral fistula, pneumonitis

## Abstract

A systematic treatment plan of controlling chronic sinusitis, optimizing systemic health, and appropriate selection of surgical technique are essential requirements for successful closure of oroantral fistula.

## Graphical abstract

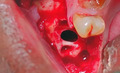

## 1 INTRODUCTION

Closure of a long‐standing oroantral fistula presents a surgical challenge. We present the case of oroantral fistula with maxillary sinusitis complicated by systemic comorbidities of advanced pulmonary disease, diabetes, and hypertension and its successful closure using a combination flap of buccal fat pad and buccal advancement flap.

The development of oroantral fistula is an uncommon event after maxillary posterior tooth extraction. Rarer still is the detection of such a fistula interdentally between maxillary posterior teeth. Other causes may include trauma, maxillary cysts and tumors, and infections. Successful closure of chronic oroantral fistula is a surgical challenge.^1^ Factors that contribute to failure of closure and wound breakdown are maxillary sinusitis which either is preexistent or occurs as a result of the communication, size of defect, failure to achieve tension‐free closure, and immunosuppression.

The various techniques used to close oroantral fistula include the buccal advancement flap, palatal rotation flap, combination flaps, bone grafts, and buccal fat pad.^2^ Occasionally, even a nonsurgical treatment modality like palatal splint is used.^3^ These techniques can be used alone or in combination, a two‐layered, or even a three‐layered closure, to improve the chances of successful fistula closure.^4^

A combination flap of buccal fat pad and buccal advancement has also been utilized.^5^ Such a technique offers a synergistic advantage by offsetting the drawbacks of the individual techniques—shrinkage with buccal fat pad and tension in flap with buccal advancement. However, there is as yet no consensus on the best technique that is to be used.^6^

## 2 CASE REPORT

**Figure 1:**
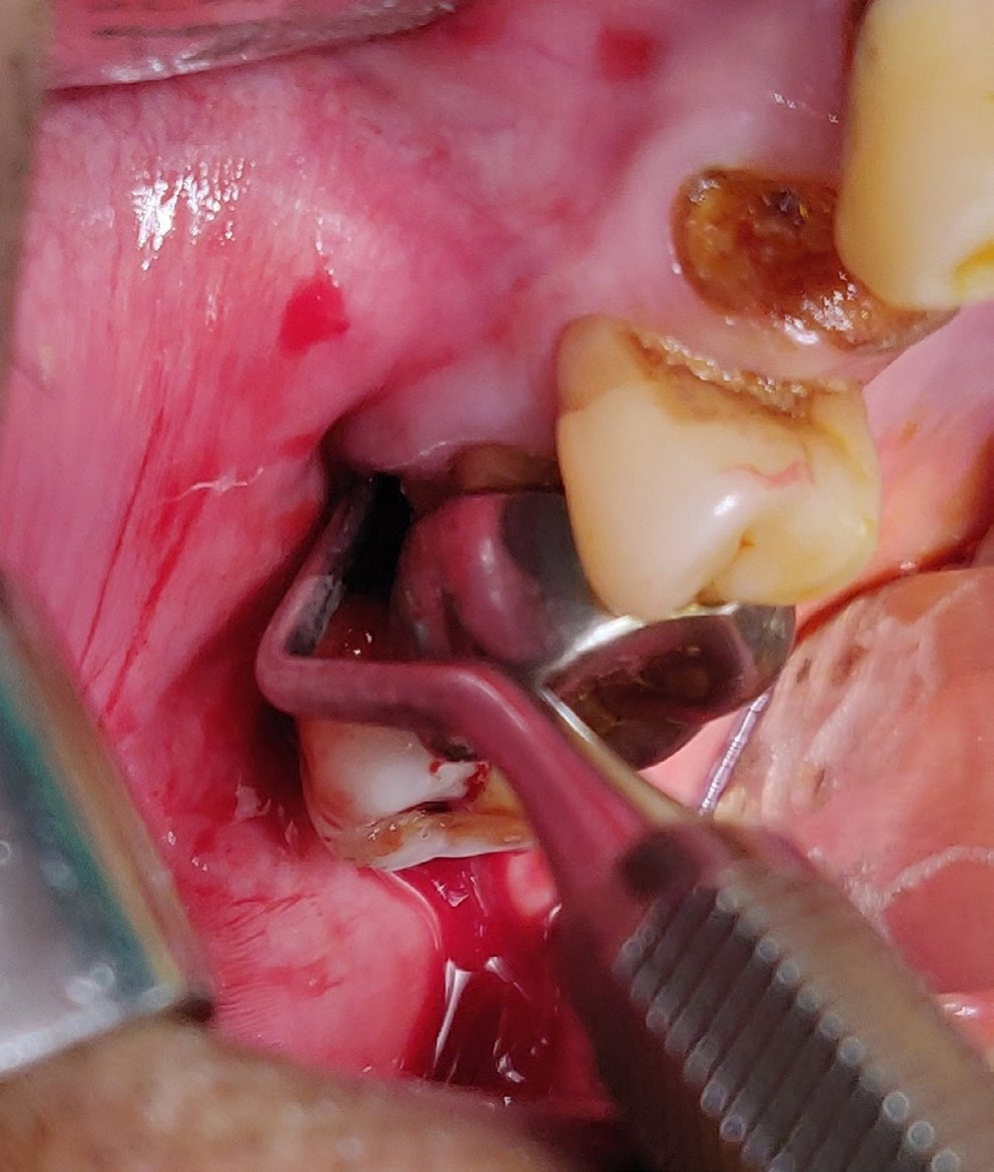
Interdental oroantral fistula

**Figure 2:**
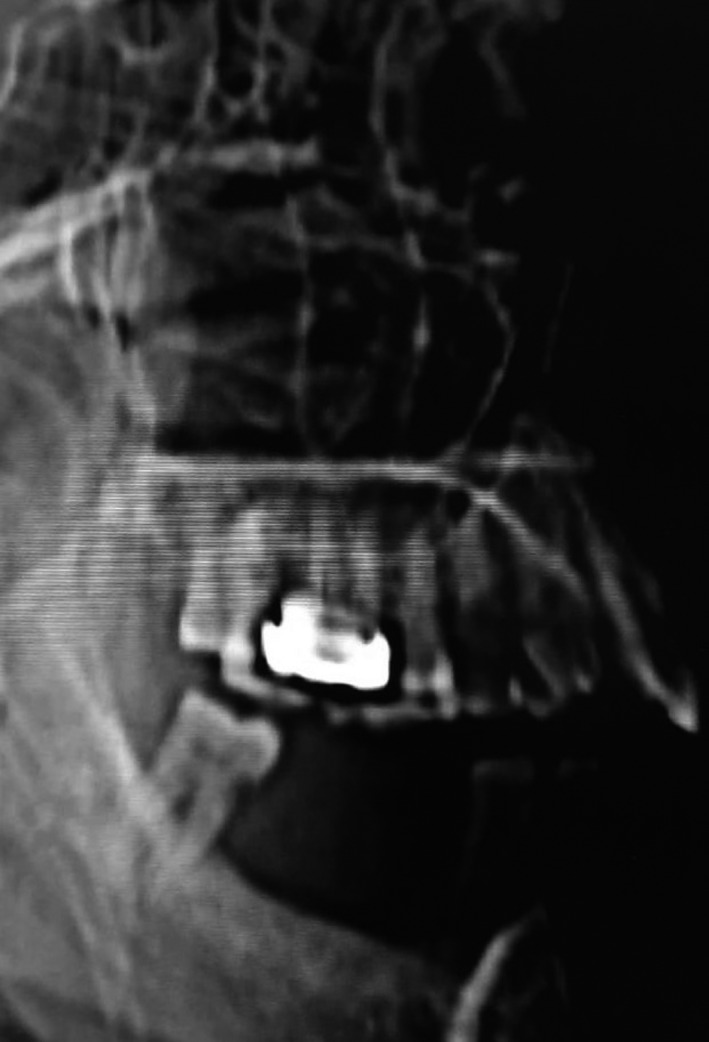
CT view depicting dentition and sinus floor

A 57‐year‐old male patient was referred with a complaint of toothache, intractable headache, and right side nasal regurgitation of fluid since 10 months. Patient revealed a history of root canal treatment of maxillary right first molar which was followed, later, by the development of nasal regurgitation of fluids. There was an oroantral fistula in the interdental area between the right maxillary first and second molars (Figure 1 and Figure 2). The teeth had poor periodontal health.

The patient was a case of chronic hypersensitivity pneumonitis which had progressed to end‐stage lung disease necessitating the use of domiciliary oxygen over last 5 years. He also had other significant comorbidities like systemic hypertension, diabetes mellitus, hypothyroidism, bipolar disorder, and pulmonary hypertension optimized on medications. The oroantral fistula compounded his health problem by contributing to acute episodes of painful maxillary sinusitis.

**Figure 3:**
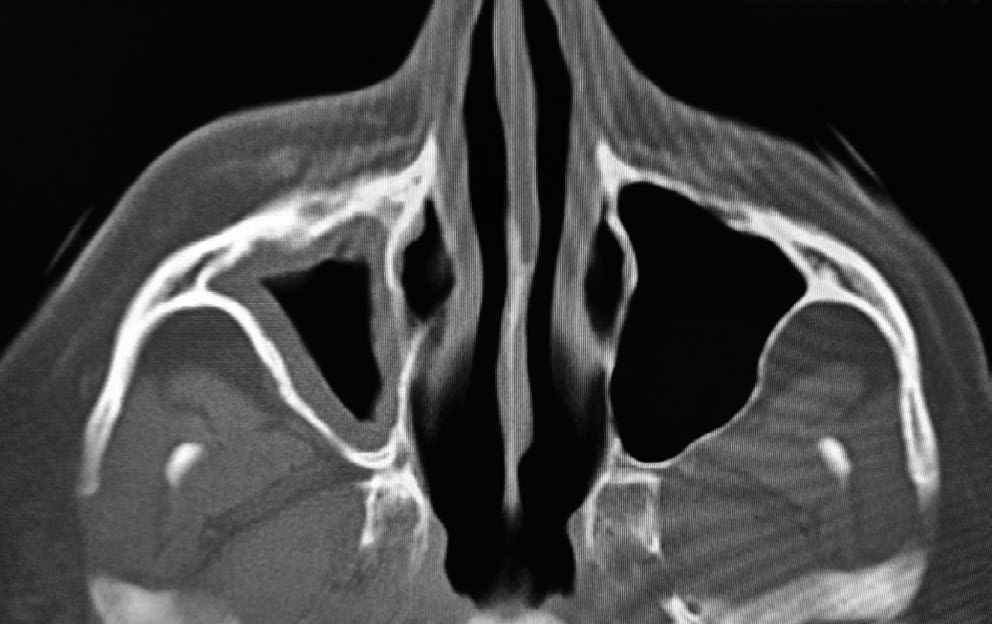
Axial section CT scan reveals maxillary sinusitis

**Figure 4:**
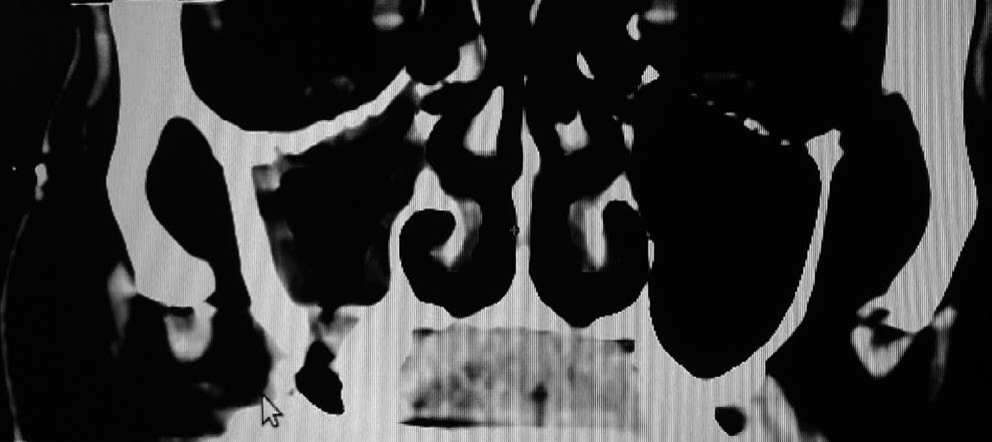
Coronal section CT scan reveals bone discontinuity in sinus floor

Computerized tomography scans revealed thickening of right maxillary sinus lining and bony defect in floor of maxillary sinus (Figure 3 and Figure 4).

A treatment plan was surgical closure of oroantral fistula. The first essential step was control of the maxillary sinusitis in consultation with otorhinolaryngologist. The patient was placed on an antral regimen of antibiotics, anti‐inflammatory drugs, nasal decongestants, and steam inhalations. The patient was then prepared for surgery under general anesthesia in consultation with pulmonologist. He was cleared for procedure with high risk.

**Figure 5:**
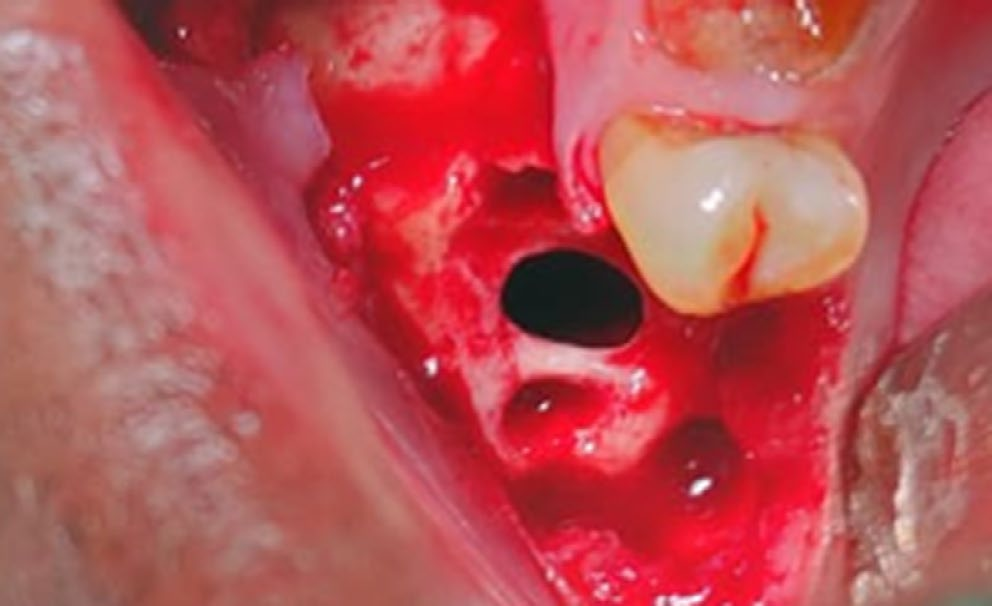
Oroantral opening visualized after tooth extraction

Intraoperatively, the first and second maxillary molars were extracted to reveal an oval opening approximately 5 × 7 mm (Figure 5). A buccal mucoperiosteal trapezoidal flap was raised and fistulous tract was excised. A distal extension of the incision in the maxillary third molar region followed by soft tissue dissection located the buccal fat pad which was carefully released from its surrounding attachments. An extension of the anterior releasing incision exposed the wall of the maxillary sinus. A bone window was prepared and the sinus lining curetted out. Food debris was recovered from the sinus. Sinus lavage was performed.

**Figure 6:**
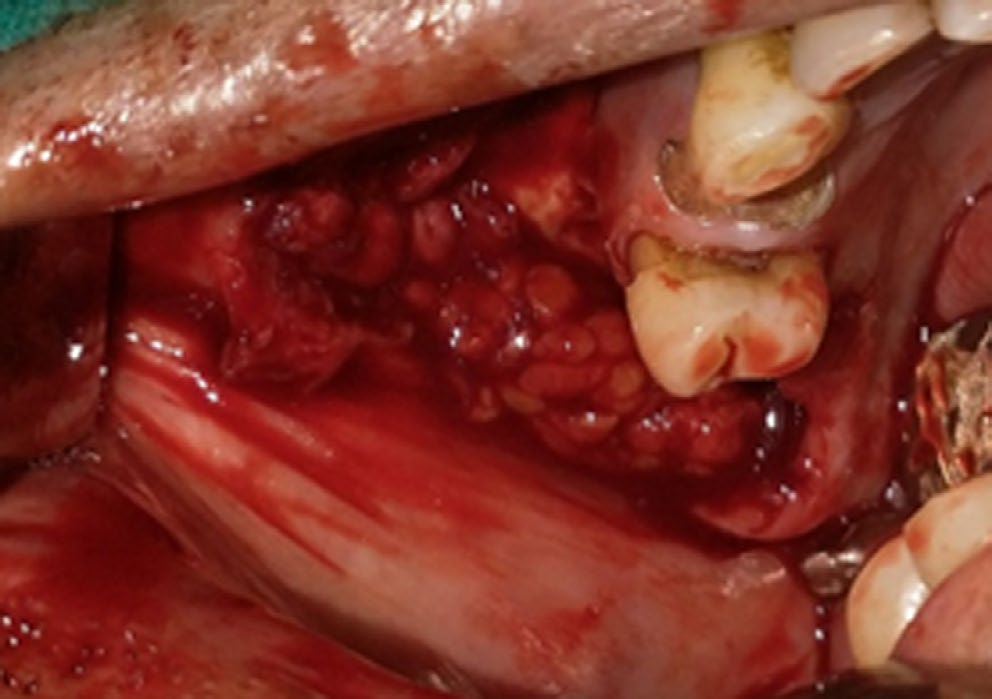
Pedicled buccal fat pad placed over defect

**Figure 7:**
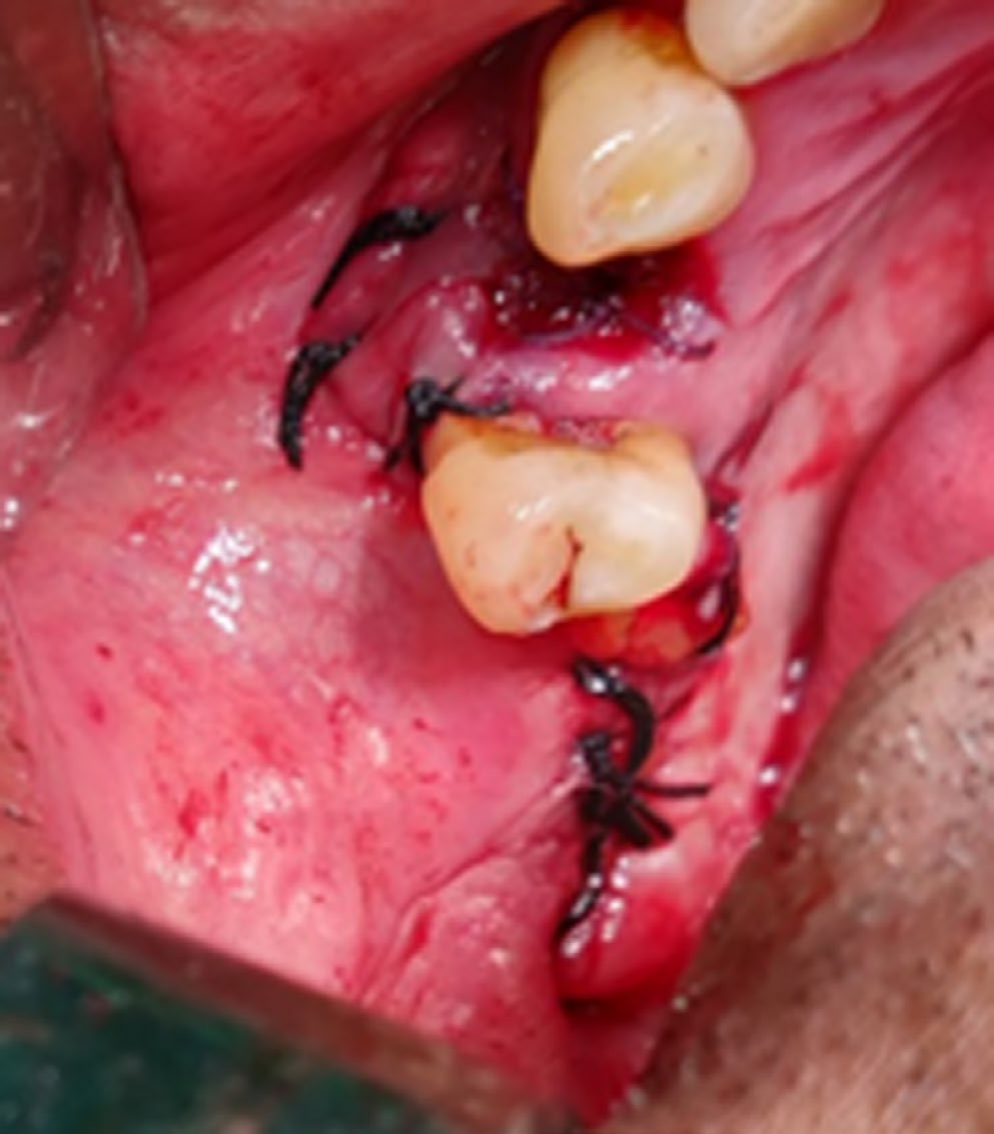
Buccal advancement flap closure

Double‐layered closure of oroantral fistula was then achieved by rotating the buccal fat pad to cover the defect and suturing it to the palatal mucoperiosteum (Figure 6). This was followed by periosteal release and advancement of the buccal flap over buccal fat pad and suturing it to the buccal fat pad and palatal mucoperiosteum (Figure 7). Postoperatively, the patient was placed on antibiotics, nasal decongestants, and steam inhalations. Appropriate monitoring and care of his pulmonary condition was provided by pulmonologist.

**Figure 8:**
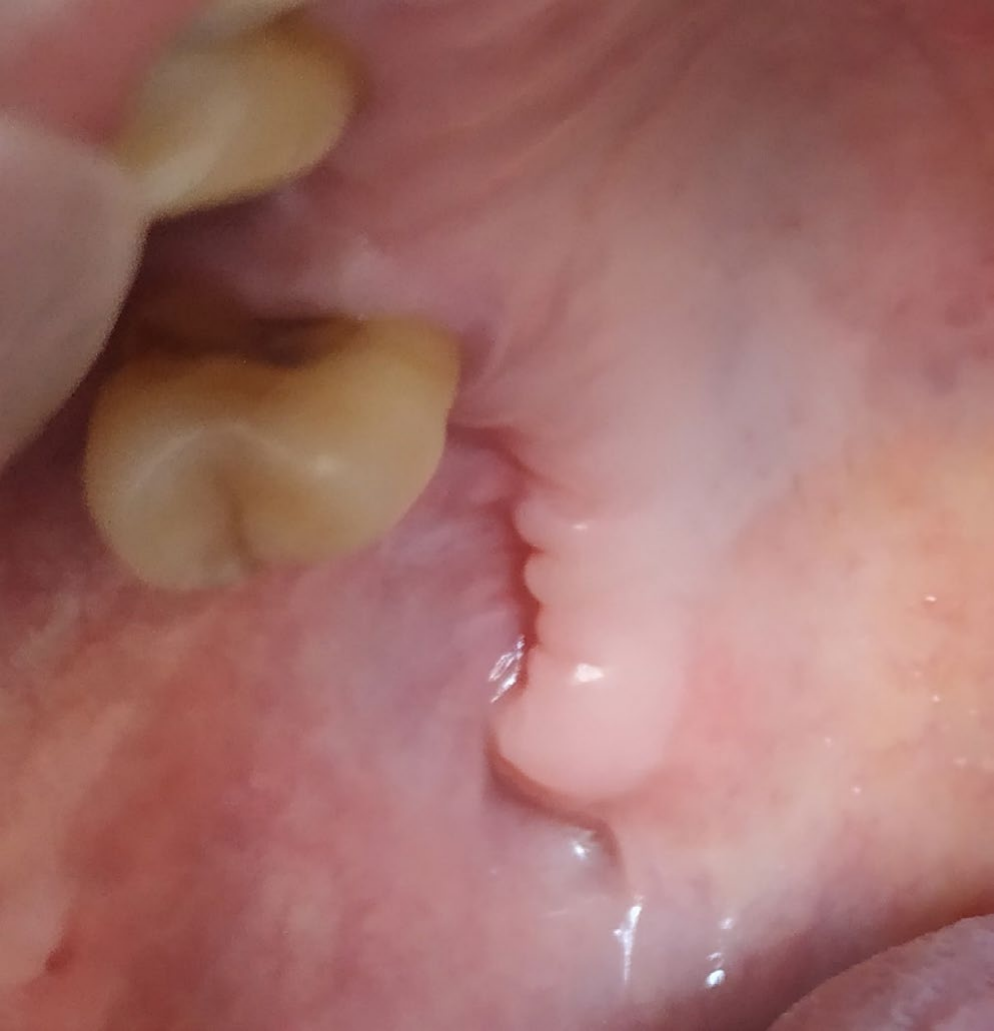
Postoperative healing

The flap healed uneventfully. Loss of buccal vestibular depth was present (Figure 8). A slight reduction in mouth opening was present in the immediate postoperative period but full mouth opening was regained subsequently. There was complete resolution of all sinus‐related symptoms.

## 3 DISCUSSION

Double‐layered closure of oroantral fistula is a technique with limited indications. This is because the buccal fat pad alone can be used. It undergoes epithelialization and is the most successful technique for fistula closure.^7^ Perforation or stretching of buccal fat pad is an indication for double‐layered closure.^8^

Systemic comorbidities influence surgical treatment plan. Preexisting pulmonary disease is a risk factor for postoperative pulmonary complications,^9^ postoperative pneumonia secondary to low lung reserves associated postoperative atelectasis, and worsening of preexisting respiratory failure. Long operating times and procedures involving high blood loss are to be avoided. Pathophysiologic changes associated with diabetes cause direct cellular damage and vascular and immune dysfunction. Postoperative complications are related to both perioperative hyperglycemia and long‐term glycemic control.^10^ Hypertension is a risk factor for cardiovascular disease and outcomes, though modifiable with appropriate medications. In this challenging medical scenario, the combination flap with good blood supply to both buccal fat pad and buccal advancement flap, proximity to recipient site permitting short surgical time, easy manipulation and minimal donor site morbidity, one flap providing protection against perforation or necrosis of the other is an appropriate surgical treatment choice.

Hence, through the case illustration, an additional indication for the use of combination flaps is a medically compromised patient, with local and systemic factors that could potentially hinder healing and in whom repeated attempts at closure is not practical. Combination flap offers greater stability and reliability.

Postoperative sequelae to be expected with this surgical procedure are pain, swelling, restriction of mouth opening, and loss of vestibular depth. Complications that have been reported to occur are partial necrosis, hemorrhage, hematoma, excessive scarring, and facial nerve injury.^11^, ^12^ In our case, the patient reported a mild limitation of mouth opening and loss of buccal vestibular depth was present. No other complications were encountered.

The occurrence of oroantral fistula interdentally between teeth is an unusual finding in this case. The different etiologies that may cause oroantral fistula with chronic sinusitis are inflammatory (infectious or noninfectious), neoplastic (primary or metastatic), odontogenic, spontaneous, iatrogenic, or persistence of infection.^13^, ^14^ In this case, after root canal therapy having been performed for a tooth in close proximity to maxillary sinus, a persistent periapical and periodontal infection with bone loss led to the formation of oroantral fistula.

To conclude, though an inset buccal fat pad graft will epithelialize and does not require coverage, in specific instances as discussed above, surgical plan for combination flap with coverage of buccal fat pad graft should be considered. A systematic treatment approach of controlling local adverse factors of infection and sinusitis, optimizing general health, and choice of surgical technique tailored to individual patient remain the cornerstones of successful oroantral fistula closure.

## CONFLICT OF INTEREST

None declared.

## AUTHOR CONTRIBUTIONS

AR: performed the surgical procedure, prepared, reviewed, edited and submitted the manuscript. VA: medical management of patient, reviewed and edited the manuscript.
